# Comparative Study of Chikungunya Only and Chikungunya-Scrub Typhus Coinfection in Children: Findings from a Hospital-Based Observational Study from Central Nepal

**DOI:** 10.1155/2021/6613564

**Published:** 2021-04-20

**Authors:** Santosh Pathak, Nagendra Chaudhary, Prativa Dhakal, Sanjay Ray Yadav, Binod Kumar Gupta, Om Prakash Kurmi

**Affiliations:** ^1^Department of Pediatrics, Chitwan Medical College, Bharatpur, Nepal; ^2^Department of Pediatrics, Universal College of Medical Sciences, Bhairahawa, Nepal; ^3^Department of Nursing, Chitwan Medical College, Bharatpur, Nepal; ^4^Department of Biochemistry, Chitwan Medical College, Bharatpur, Nepal; ^5^Centre for Population Health Research, Bhairahawa, Nepal

## Abstract

**Objectives:**

Chikungunya and scrub typhus infection are important causes of undifferentiated fever in tropical zones. The clinical manifestations in both conditions are nonspecific and often overlap. This study compares the clinical manifestations and the outcome of chikungunya with chikungunya-scrub typhus coinfection in children.

**Methods:**

A hospital-based observational study was conducted in children below 15 years of age over 16-month duration in 2017-2018. Chikungunya was diagnosed by IgM ELISA. All positive chikungunya cases were subjected to scrub typhus testing, dengue testing, leptospira testing, and malaria testing. Clinical manifestations and outcomes of all patients were recorded.

**Results:**

Out of the 382 admitted cases with fever, 11% (*n* = 42) were diagnosed with chikungunya, and the majority (*n* = 30, 71.4%) were male. Among the 42 chikungunya cases, 17 (40.5%) tested positive for scrub typhus and one positive for falciparum malaria. Out of a total of 42 chikungunya cases, myalgia, nausea/vomiting, headache, abdominal pain, lymphadenopathy, hepatomegaly, splenomegaly, and edema were 81%, 73.8%, 66.7%, 64.3%, 59.5%, 52.4%, 40.5%, and 38.1%, respectively. Besides, altered sensorium (31%), jaundice (26.2%), dry cough (21.4%), shortness of breath (19%), and seizures (16.7%) were other clinical manifestations present in this group of children. Patients with chikungunya-scrub typhus coinfection reported headaches, pain in the abdomen, dry cough, shortness of breath, seizures, and splenomegaly, significantly more (*p* value < 0.05) compared to those with chikungunya only. Thirteen (31%) children developed shock, five in the chikungunya group and eight in the chikungunya-scrub typhus coinfection group. Six children in the coinfection group received inotrope. Among the chikungunya-only cases, 22 recovered and one died, whereas in the chikungunya-scrub typhus coinfection group, fourteen recovered and three died.

**Conclusions:**

Both the chikungunya and scrub typhus coinfection groups shared many similar clinical manifestations. In children, coinfection with scrub typhus often leads to modification of the clinical profile, complications, and chikungunya outcome.

## 1. Introduction

Chikungunya and scrub typhus are important causes of undifferentiated fever in tropical and subtropical regions. Chikungunya fever is an arthropod vector-borne infectious disease caused by the chikungunya arbovirus (CHIKV) and transmitted to humans through the bite of *Aedes* mosquitoes (*Aedes aegypti* and *A. albopictus*), whereas scrub typhus is a clinical disease caused by a zoonotic obligate intracellular, gram-negative bacteria (*O*. *tsutsugamushi*), transmitted by the bite of trombiculid mite, the chigger [1, 2].

The clinical presentations of both chikungunya and scrub typhus are nonspecific, and both clinical entities are encountered as a vital differential of undifferentiated fever in tropical zones. This often creates challenges for accurate diagnosis and treatment in poor-resource settings. Emerging tropical diseases like chikungunya and scrub typhus in children are underdiagnosed in low-income countries (LICs) as the presentation, awareness, and index of suspicion are neglected among clinicians. Lack of reliable diagnostic facilities and their availability in all Nepal centres are also important reasons for the disease's underdiagnosis, often delaying specific treatment with increased case fatality rates [2, 3].

Both chikungunya and scrub typhus can present with fever, myalgia, hepatosplenomegaly, and lymphadenopathy [1–3]. The management of monoinfection with chikungunya and coinfections varies [2, 4]. Therefore, a proper understanding of clinical features and complications, along with appropriate laboratory investigations, is necessary. Any delay in the diagnosis and differentiation of such conditions could increase the severity of symptoms and higher mortality. Although a recent study from India by Rao et al. found a higher rate of coinfection with malaria and dengue in patients (1-70 years) with chikungunya and scrub typhus, respectively [5], so far to our knowledge, no study has compared the chikungunya-scrub typhus coinfection. Therefore, this hospital-based study was aimed at comparing the clinical profiles and outcomes of chikungunya only and chikungunya-scrub typhus coinfection.

## 2. Methods

### 2.1. Hospital Setting, Patient Selection, and Diagnosis

A prospective observational study was conducted at Chitwan Medical College Teaching Hospital (CMC-TH), a tertiary care referral teaching hospital in Nepal's central region, over 16 months (1 May 2017-30 September 2018) aged 0-15 years. All suspected cases with fever (*n* = 382) but without any identifiable infection (undifferentiated fever) along with the presence of one or more of the following clinical features (joint pains, rashes, edema, hepatosplenomegaly, lymphadenopathy, and eschar) were subjected to a serological test for chikungunya and scrub typhus.

Serological diagnosis of chikungunya was performed by IgM Elisa (Euroimmun Medizinische Labordiagnostika AG, 23560 Lubeck, Germany) and scrub typhus by IgM ELISA test (In BiOS International, Inc. Seattle USA). Common infectious conditions that could clinically mimic chikungunya were ruled out by performing the following tests: peripheral smear and rapid antigen test for malaria, dengue (NS1 antigen and IgM antibody) test, and urine and blood cultures as per clinical aspect. Leptospira serology was performed when clinically indicated. Cardiac evaluation (echocardiography and CPK-MB) and cerebrospinal fluid (CSF) analysis was performed for selected cases with suspected myocarditis or meningoencephalitis, respectively.

All admitted children (*n* = 382) started with conservative treatment, intravenous ceftriaxone (100 mg/kg/day) and amikacin (15 mg/kg/day) on admission. Intravenous antibiotics were discontinued once the diagnosis of chikungunya was confirmed. Children diagnosed with scrub typhus were treated with a 7–10-day course of doxycycline (5–7 mg/kg/day twice daily). In contrast, those showing poor response to doxycycline (fever persisting even after 72 hours of therapy) were treated with oral or intravenous azithromycin for five days.

### 2.2. Variables

All the clinical data, including the duration of fever, associated symptoms, vital signs, and the general and systemic examination findings, were recorded in a predesigned proforma. Data regarding age, sex, and residential area were collected. Complete blood counts, chest X-rays, renal function test (urea, creatinine), liver function test (serum bilirubin, aspartate transaminase, alanine transaminase, serum albumin, total protein, and alkaline phosphatase), and urinalysis were performed at the time of presentation for all cases and were repeated where necessary.

### 2.3. Case Definitions

- Acute kidney injury: rise in serum creatinine at least 0.3 mg/dl or 50% higher than the baseline within a 24- to 48-hour period or a reduction in urine output to 0.5 ml/kg per hour for more than 6 hours [2]
- Meningitis: child having altered sensorium along with meningeal signs and/or seizures plus CSF study showing raised protein and lymphocytic/neutrophilic cytology with normal or low sugar [2]
- Acute hepatitis: elevation of serum transaminases more than two times the normal upper limit [2]

### 2.4. Statistical Analysis

The data were analysed using SPSS software, version 16.0 (SPSS Inc., Chicago, IL). Descriptive statistics in terms of frequency, percentage, mean, and standard deviation were calculated. A chi-square test was used to test the independence of two categorical variables with a *p* value of less than 0.05 considered statistically significant.

## 3. Results

Out of the 382 admitted cases with undifferentiated fever, 11% (*n* = 42) were diagnosed with chikungunya with male predominance (71.4%, *n* = 30) but without any distinct age patterns (33.3%, 23.8%, and 30.9% in the age groups 0-5 years, 6-10 years, and 11-15 years, respectively) (Table 1). Among the 42 diagnosed cases with chikungunya, 17 children (40.5%) also tested positive for scrub typhus, and one child had falciparum malaria.

### 3.1. Chikungunya with or without Scrub Typhus (*n* = 42)

More than two-thirds (71.4%, *n* = 30) of the cases presented with a history of fever less than 7 days and 28.6% (*n* = 12) with seven days or more. The proportions of cases that reported myalgia, nausea/vomiting, headache, abdominal pain, lymphadenopathy, hepatomegaly, splenomegaly, and edema were 81%, 73.8%, 66.7%, 64.3%, 59.5%, 52.4%, 40.5%, and 38.1%, respectively. The presence of clinical manifestations like headache, abdominal pain, splenomegaly, dry cough, shortness of breath, and seizures was predominant (*p* < 0.05) in the chikungunya-scrub typhus coinfection group when compared with the chikungunya-only group (Table 2). The laboratory parameters of chikungunya cases, chikungunya-scrub typhus coinfection, and chikungunya with or without scrub typhus are reported in Table 3. All had elevated mean liver enzyme (SGOT/SGPT) levels regardless of with or without scrub typhus.

### 3.2. Chikungunya Only (*n* = 25)

The majority of children (56%, *n* = 14) were in the age group 0-5 years, whereas the rest were in 6-15 years. 76% (*n* = 19) of the children presented with fever for less than seven days and the rest (24%, *n* = 6) for seven days or more (Table 2). The most common clinical presentations of children with chikungunya-only cases were myalgia (76%, *n* = 19), nausea/vomiting (64%, *n* = 16), abdominal pain (52%, *n* = 13), headache (48%, *n* = 12), lymphadenopathy (48%, *n* = 12), and hepatomegaly (44%, *n* = 11). The mean systolic and diastolic blood pressures were 93.8 ± 7.7 mmHg and 61.4 ± 5 mmHg, respectively. Three children required fluid bolus, and two received inotrope.

### 3.3. Chikungunya and Scrub Typhus Coinfection (*n* = 17)

Seven children (41.2%) in the age group 6-10 years and 29.4% in every 0-5 and 11-15 years had chikungunya and scrub typhus. 64.7% (*n* = 11) of the children presented with a fever duration of fewer than seven days. Some of the clinical manifestations in this group were headache (94.1%, *n* = 16), joint pain (88.2%, *n* = 15), nausea/vomiting (88.2%, *n* = 15), abdominal pain (82.4%, *n* = 14), lymphadenopathy (76.4%, *n* = 13), splenomegaly (70.6%, *n* = 12), hepatomegaly (64.7%, *n* = 11), and edema (53%, *n* = 9). Six children received inotropic support, and two received fluid bolus only (Table 2).

### 3.4. Complications

Thirteen children developed shock. Among those who developed shock, eight received inotropic support and five fluid boluses when presented with hypotension. Eleven children developed acute hepatitis, and three children had acute kidney injury, whereas four children had meningitis.

### 3.5. Outcome

Out of the 42 cases, 36 children (85.7%) recovered well (22 from the chikungunya-only group and 14 from those with chikungunya and scrub typhus), and 4 (9.5%) died (one from the chikungunya-only group and three from those with chikungunya and scrub typhus). The two cases' outcome was unknown as they went on leave against medical advice (LAMA) and could not be followed up.

## 4. Discussion

The presentation of clinical manifestations can vary in chikungunya and chikungunya-scrub typhus coinfection. The chikungunya-scrub typhus coinfection group reported significantly more headaches, abdominal pain, dry cough, shortness of breath, seizures, and splenomegaly than the chikungunya-only group in the present study.

The proportion of males was more than females, both in the “chikungunya-only” and chikungunya-scrub typhus coinfection groups, possibly due to higher involvement in male children's outdoor activities. A recent systematic review reported that chikungunya is more common in 20-40 years of age [6]. Similarly, a study conducted in India in 2006 found a seropositivity rate of 4.37% for chikungunya antibodies. It was more in 51-55 age groups and none in the younger age groups suggesting the lack of herd immunity to chikungunya virus in the younger population [7]. However, in contrast to the above two studies, our findings indicate that the proportion of chikungunya in 0-5-year-old children was higher than 6-14 years, as reported in another recent study with higher severe disease in infants and neonates [8].

Clinical features in chikungunya and scrub typhus are nonspecific and broad and can involve body systems [6]. Children can present with fever, body ache, joint pain, edema, headache, nausea/vomiting, and rashes. These clinical presentations can be present in many tropical infections like dengue, scrub typhus, leptospirosis, and malaria [3]. In the present study, all the children with chikungunya only and chikungunya-scrub typhus coinfection had a fever. A previous study by Saleem et al., in patients with dengue and scrub typhus coinfection, also reported fever in all the cases [9]. Still, there is no previous published literature to compare chikungunya-scrub typhus coinfection clinical features to the best of our knowledge.

The most common clinical presentation of febrile children with “chikungunya-only” cases was joint pain, nausea/vomiting, abdominal pain, headache, lymphadenopathy, and hepatomegaly. In contrast, headache, joint pain, nausea/vomiting, abdominal pain, lymphadenopathy, splenomegaly, hepatomegaly, and edema were the common manifestations seen in chikungunya-scrub typhus coinfection in the present study. A study conducted in India in 2006 reported all the cases of chikungunya had a fever and joint pain, and 55.1% of the patients reported headaches [10]. Other studies in children with chikungunya have demonstrated joint pain in 30-50% of cases and headache in 15% of cases [1, 11]. In the present study, children with chikungunya only had joint pain in 76% of cases and headache in 48% of cases. Joint pain was predominantly more in the chikungunya group than the coinfection group, whereas the headache was more in the coinfection group in the present study.

A 2005-2006 outbreak in Reunion found skin exanthem in 48% of cases [12]. In a study conducted by Ritz et al., about 60% of chikungunya patients had skin rashes. However, an Indian study reported as low as 31% [10, 11]. In the present study, skin rashes were almost negligible in both the chikungunya and coinfection groups. Eschar, a pathognomonic sign in scrub typhus, was present in 17.6% (3 out of 17) in the coinfection group, and none in the chikungunya-only group, slightly higher than our previous study that reported 12% of patients had eschar [2].

A recent systematic review reported an increase in the neurological disease in chikungunya patients [13] with seizures, acute encephalopathy, and meningoencephalitis ranging from 14 to 32% in children [1, 11]. Similarly, our previous study in Nepalese children with scrub typhus reported that 11.8% of children had seizures [2]. In the present study, 6% of the chikungunya group had seizures, whereas over one-third (35.2%) in the coinfection group had seizures. This suggests that seizure is an important finding, and chikungunya alone or chikungunya-scrub typhus coinfection should be considered as an important differential in children presenting with fever and seizures.

Alteration in children's laboratory parameters with chikungunya and scrub typhus has been documented in different studies [1, 2, 11]. We found a significant alteration of serum transaminases in the chikungunya and chikungunya-scrub typhus coinfection groups in the present study.

Chikungunya can lead to complications like sepsis, shock, liver failure, and encephalopathy. In a study conducted on 49 children with chikungunya by Sharma et al., 13 children had severe disease, with 11 having severe sepsis with shock and 2 with acute liver failure [14]. In the present study, most children with shock in the chikungunya group responded well to fluid boluses, whereas the majority required inotropes in the chikungunya-scrub typhus coinfection group. The mortality in the coinfection group was more in comparison to the chikungunya-only group. Although chikungunya has a mild course, few of them can have life-threatening complications. Coinfection of chikungunya with scrub typhus may further increase the frequency, worsen the complications, and lead to higher mortality. A recent systematic review concluded the disease to be fatal in children and the elderly, even in the absence of comorbidities [6].

The present study has a few limitations. As the sample size was small, further studies with large samples should be compared in both the chikungunya and coinfection groups. Secondly, our study is a single-centre study which may not depict the population as a whole.

## 5. Conclusion

Clinical manifestations, complications, and outcomes of chikungunya in children can vary when coinfected with scrub typhus. Therefore, a high index of suspicion is necessary to think of coinfection, mainly depending on the varied clinical presentation range.

## Figures and Tables

**Table 1:** Demographic data.

	Only chikungunya *N* = 25	Chikungunya and scrub typhus *N* = 17	Total cases *N* = 42
Sex			
Male	19 (63.3)	11 (36.7)	30 (71.4)
Female	6 (50.0)	6 (50.0)	12 (28.6)
Age group (in years)			
0 to 5	14 (73.7)	5 (26.3)	19 (33.3)
6 to 10	3 (30.0)	7 (70.0)	10 (23.8)
11 to 15	8 (61.5)	5 (38.5)	13 (30.9)

**Table 2:** Signs and symptoms.

Signs and symptoms	Only chikungunya *N* = 25	Chikungunya and scrub typhus *N* = 17	Total cases *N* = 42
Fever			
<7 days	19 (63.3)	11 (36.7)	30 (71.4)
≥7 days	6 (50.0)	6 (50.0)	12 (28.6)
Joint pain	19 (55.9)	15 (44.1)	34 (81.0)
Nausea/vomiting	16 (51.6)	15 (48.4)	31 (73.8)
Headache^∗^	12 (42.9)	16 (57.1)	28 (66.7)
Lymphadenopathy	12 (48.0)	13 (52.0)	25 (59.5)
Abdominal pain^∗^	13 (48.1)	14 (51.8)	27 (64.3)
Hepatomegaly	11 (50.5)	11 (50.0)	22 (52.4)
Splenomegaly^∗^	5 (29.4)	12 (70.6)	17 (40.5)
Edema	7 (43.7)	9 (56.2)	16 (38.1)
Jaundice	4 (36.4)	7 (63.6)	11 (26.2)
Dry cough^∗^	2 (22.2)	7 (77.8)	9 (21.4)
Shortness of breath^∗^	1 (12.5)	7 (87.5)	8 (19.0)
Seizure^∗^	1 (14.3)	6 (85.7)	7 (16.7)
Altered sensorium	6 (46.1)	7 (53.8)	13 (31.0)
Eschar	0 (0.0)	3 (100.0)	3 (7.1)
Rashes	1 (50.0)	1 (50.0)	2 (4.8)
Blood pressure (mean ± SD)			
Systolic (mmHg)	93.8 ± 7.7	88.8 ± 9.3	91.8 ± 8.6
Diastolic (mmHg)	61.4 ± 5.7	55.8 ± 12.8	59.1 ± 9.5
Hypotension/shock			
Fluid bolus only (*n* = 5)	3 (60.0)	2 (40.0)	5 (38.5)
At least one inotrope (*n* = 8)	2 (25.0)	6 (75.0)	8 (61.5)

Values are number and percentages; ^∗^*p* < 0.05.

**Table 3:** Clinicolaboratory parameters.

	Only chikungunya (*N* = 25) Mean (min–max)	Chikungunya and scrub typhus (*N* = 17) Mean (min–max)	Total cases (*N* = 42) Mean (min–max)
Hemoglobin (gm/dl)	11.0 (8.5–13.5)	10.9 (8.4–13.9)	11.0 (8.4–13.9)
PCV	33.6 (25.0–40.7)	32.7 (25.1–41.0)	33.2 (25.0–41.0)
Total count (cells/mm^3^)	8032 (2300–21200)	13171 (2300–3500)	10112 (2300–35000)
Neutrophil (%)	57 (1.0–88)	69 (38–87)	61 (17–88)
Lymphocytes (%)	37 (1–71)	30 (9–60)	34 (1–71)
Platelet counts (cells/mm^3^)	195760 (77000–497000)	131294 (14000–378000)	169667 (14000–497000)
Bilirubin total (mg/dl)	1.4 (0.6–7.9)	1.2 (0.4–2.7)	1.3 (0.4–7.9)
SGOT (IU/l)	374.7 (14–5852)	193.9 (17–513)	301.5 (14–5852)
SGPT (IU/l)	309.2 (14–4983)	139.0 (21–512)	240.3 (14–4983)
Serum albumin (gm/dl)	3.5 (2.5–4.7)	2.9 (1.9–4.8)	3.3 (1.9–4.8)
PT control (secs)	16.2 (12–39)	17.3 (12–24)	16.7 (12–39)
INR	1.3 (1–3.2)	1.4 (1–2.2)	1.3 (1–3.2)
aPTT	30.2 (20–48)	35.1 (18–55)	32.2 (18–55)
Urea (mg/dl)	24 (10–68)	46.9 (13–187)	33.3 (10–187)
Creatinine (mg/dl)	0.6 (0.3–1.6)	0.7 (0.3–2.6)	0.6 (0.3–2.6)
Sodium (meq/l)	137.6 (132–148)	134.0 (124–140)	136.1 (124–148)
Potassium (meq/l)	9.2 (2.7–139)	4.0 (2.9–5.5)	7.1 (2.7–139)
CPK-MB (U/l)	25 (10–117)	29 (11–82)	26 (10–117)
CRP quantitative (mg/dl)	34.4 (3.2–184.1)	63.7 (0.3–183.5)	46.3 (0.3–184.1)

PCV: packed cell volume; SGOT: serum glutamic-oxaloacetic transaminase; SGPT: serum glutamic-pyruvic transaminase; PT: prothrombin time; INR: international normalized ratio; aPTT: activated partial thromboplastin time; CPK-MB: creatine phosphokinase (MB form); CRP: C-reactive protein.

## Data Availability

Data has been provided in Methods of the manuscript. If required, data can be obtained from the corresponding author on reasonable request.

## Abbreviations

CHIKV: Chikungunya arbovirus
LIC: Low-income countries
PCV: Packed cell volume
SGOT: Serum glutamic-oxaloacetic transaminase
SGPT: Serum glutamic-pyruvic transaminase
PT: Prothrombin time
INR: International normalized ratio
aPTT: Activated partial thromboplastin time
CPK-MB: Creatine phosphokinase (MB form)
CRP: C-reactive protein.
